# Evaluation of diagnostic and treatment approaches to acute dyspnea in a palliative care setting among medical doctors with different educational levels

**DOI:** 10.1007/s00520-022-06996-6

**Published:** 2022-03-25

**Authors:** Klaus Hackner, Magdalena Heim, Eva Katharina Masel, Gunther Riedl, Michael Weber, Matthäus Strieder, Sandra Danninger, Martin Pecherstorfer, Gudrun Kreye

**Affiliations:** 1 Karl Landsteiner University of Health Sciences, Krems, Austria; 2 Department of Pneumology, University Hospital Krems, Krems, Austria; 3 Clinical Division of Palliative Medicine, Department of Internal Medicine I, Medical University Vienna, Vienna, Austria; 4 Department for Anesthesia and Intensive Care, Landesklinikum Baden-Mödling, Baden, Austria; 5 Clinical Division of Palliative Medicine, Department of Internal Medicine II, University Hospital Krems, Mitterweg 10, 3500 Krems, Austria

**Keywords:** Palliative care, Cancer, Medical doctors, Dyspnea, Case report

## Abstract

**Background:**

Dyspnea is common in patients with advanced cancer. Diagnostic procedures in patients with dyspnea are mandatory but often time-consuming and hamper rapid treatment of the underlying refractory symptoms. Opioids are the first-line drugs for the treatment of refractory dyspnea in palliative care patients with advanced lung cancer.

**Methods:**

To evaluate the knowledge levels of medical doctors with different educational levels on the diagnosis of and treatment options for dyspnea in patients with advanced lung cancer in a palliative care setting, a case report and survey were distributed to physicians at the University Hospital Krems, describing acute dyspnea in a 64-year-old stage IV lung cancer patient. A total of 18 diagnostic and 22 therapeutic options were included in the survey. The physicians were asked to suggest and rank in order of preference their diagnosis and treatment options. Statistical analyses of the data were performed, including comparison of the responses of the senior doctors and the physicians in training.

**Results:**

A total of 106 surveys were completed. The respondents were 82 senior physicians and 24 physicians in training (response rates of 86% and 80%, respectively). Regarding diagnostic investigations, inspection and reading the patient’s chart were the most important diagnostic tools chosen by the respondents. The choices of performing blood gas analysis (*p* = 0.01) and measurement of oxygen saturation (*p* = 0.048) revealed a significant difference between the groups, both investigations performed more frequently by the physicians in training. As for non-pharmacological treatment options, providing psychological support was one of the most relevant options selected. A significant difference was seen in choosing the option of improving a patient’s position in relation to level of training (65.9% senior physicians vs. 30.4% physicians in training, *p* = 0.04). Regarding pharmacological treatment options, oxygen application was the most chosen approach. The second most frequent drug chosen was a ß-2 agonist. Only 9.8% of the senior physicians and 8.7% of the physicians in training suggested oral opioids as a treatment option, whereas intravenous opioids were suggested by 43.9% of the senior physicians and 21.7% of the physicians in training (*p* = 0.089). For subcutaneous application of opioids, the percentage of usage was significantly higher for the physicians in training than for the senior physicians (78.3% vs. 48.8%, *p* = 0.017, respectively).

**Conclusion:**

The gold standard treatment for treating refractory dyspnea in patients with advanced lung cancer is opioids. Nevertheless, this pharmacological treatment option was not ranked as the most important. Discussing hypothetical cases of patients with advanced lung cancer and refractory dyspnea with experienced doctors as well as doctors at the beginning of their training may help improve symptom control for these patients.

**Supplementary Information:**

The online version contains supplementary material available at 10.1007/s00520-022-06996-6.

## Introduction

Dyspnea is one of the most distressing symptoms in patients with advanced cancer. The reported prevalence of dyspnea ranges from 19 to 64% in heterogeneous cancer entities [1, 2]. However, in the last weeks of life, the prevalence of dyspnea increases from 49 to 64% [3, 4].

The first-line and gold standard for managing dyspnea in patients with advanced cancer is opioids [5–8]. Nevertheless, respiratory depression remains a major concern for many physicians, which may lead to reluctance to use opioids in these patients.

### Diagnostic procedures for dyspnea

**Fig. 1:**
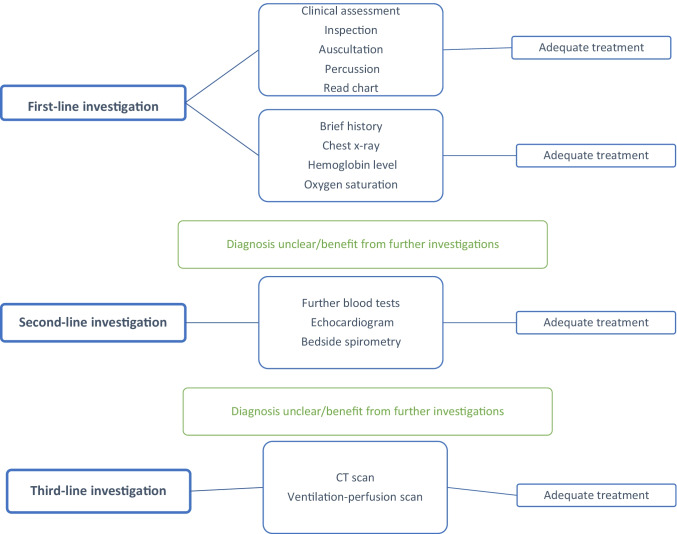
Investigations in advanced cancer patients with acute dyspnea. Adapted from Chan K.-S., Sham, M., Tse, D et al. (2005). Palliative medicine in malignant respiratory disease. The Oxford textbook of palliative care (pp. 587–618). Editors: N. Cherny, M. Fallon, S. Kaasa, R.K. Portenoy, DC Currow. Oxford University Press

Before adequate treatment is offered to advanced cancer patients with severe dyspnea, the underlying cause of breathlessness must be identified. Selected diagnostic procedures are compulsory before starting treatment (Fig. 1) [9]. First, a comprehensive clinical investigation and assessment of the patient is needed, including inspection, auscultation, and percussion [10]. After first clinical assessment, the patient might immediately benefit from adequate purposive treatment. Further investigations include evaluation of the medical history and the current problem, capturing the main facts from the patient’s chart. If necessary, chest x-ray, measuring oxygen saturation, and assessing the hemoglobin level are the diagnostic procedures that should follow [9]. In cases of acute dyspnea, assessing the patient’s medical history verbally might be difficult; instead, the patient’s chart should be used to gain essential information.

Physical examination includes a quick assessment of the patient’s general condition. Obvious signs of infection, hypoxia, anemia, bronchospasm, or effusions can reveal further reasons for dyspnea. Some patients may require further second- and third-line investigations. Before performing these investigations, treatment should be initiated to alleviate the patient’s symptoms as soon as possible (Fig. 1) [9].

### Treatment of dyspnea

The primary goal of treating dyspnea in patients with advanced cancer is relief of symptoms. Some non-pharmacologic strategies may be beneficial in the management of acute dyspnea [11]. One of them is to help the patient sit upright in a chair or bed, or in front of an open window or a fan [12].

The drugs of choice as the first-line treatment in the pharmacological management of refractory dyspnea are opioids [5, 8, 13]. Many studies have shown the value of opioids for dyspnea in patients with advanced cancer [7]. Opioids can relieve dyspnea by depressing the respiratory drive and changing the patient’s perception of dyspnea [14].

Currently, there is no evidence of a consistent beneficial effect for benzodiazepines, phenothiazines, antidepressants, diuretics, or oxygen as first-line treatments. Benzodiazepines appear not to reduce dyspnea directly, but they may decrease anxiety and can therefore be used in combination with other drugs [13, 15]. Corticosteroids, bronchodilators, and other drugs are used as second- or third-line drugs [16–20]. Oxygen therapy is still considered controversial, as few data support its use in non-hypoxic patients [21]. In daily practice, supplemental oxygen can be considered in patients with an oxygen saturation of less than 90% [22, 23].

Although there are guidelines on how to assess dyspnea in advanced cancer patients as well as treatment guidelines as listed above, many centers including our institute lack an evidence-based policy about how to assess and treat refractory dyspnea in patients with advanced lung cancer. Anecdotal observations by one of the authors (G.K.) led to the impression that physicians in training would perform more investigations and apply less opioids. Vice versa, observations by one of the authors (G.K.) also gave the impression that senior physicians would perform less investigations and are less likely reluctant to prescribe opioids. To gain objective information on these anecdotal observations, we created a case report about a lung cancer patient with refractory dyspnea. Because the observer (G.K.) had no proof whether her observations were correct or not, the authors decided to evaluate this observation by means of a hypothetical case report with a questionnaire containing guideline-recommended diagnosis and treatment options.

When starting medical practice, young doctors can find it difficult to use drugs with which they have little experience and which are still associated with approaching death, such as opioids. Observations in everyday clinical practice show that physicians with less practical experience are more likely to order examinations and use causal therapies for dyspnea such as betamimetics, corticosteroids, or diuretics. Opioid therapy is often viewed as a last resort and a therapy to be used just before death [24]. Opiophobia makes clinicians reluctant to prescribe and their patients reluctant to take opioids that might provide significant improvements in quality of life [25]. Experienced physicians may have a different approach because they have more often treated people with refractory symptoms and might have had a history of using opioids successfully. However, while physicians are mostly willing to prescribe opioids for breathlessness in the last days or weeks of life, they are often reluctant to prescribe opioids to those earlier in their disease trajectory [26]. This was investigated by using questionnaire by Hadjiphilippou et al., not differentiating among physicians with different clinical experience. In their study, doctors were aware of the use of opioids for refractory dyspnea and reported a willingness to prescribe opioids for this symptom. However, fears about side effects were prevalent.

The aim of this study was to evaluate attitudes toward diagnostic and treatment approaches, in this case report of a patient with endstage lung cancer and acute dyspnea among physicians with different clinical experience.

## Material and methods

A case report about a Union for International Cancer Control (UICC) stage IV lung cancer patient with massive pulmonary disease progression was distributed to MDs (senior physicians and physicians in training) (see Appendix). The MDs were asked to read the case report and imagine a scenario where they were contacted by a nurse because the patient was suffering from acute worsening of dyspnea. After reading the case report, the MDs were asked to rank their preferred diagnostic procedures and treatment options. To ensure that the MDs understood how to rank the diagnosis and treatment options, a recipe for how to bake a cake was provided as a model for consecutive options that should be ranked. The MDs were asked to read the recipe before ranking their decisions about the case report (see Appendix).

A detailed literature review was used to include all possible diagnostic procedures and treatment options for patients with advanced cancer and dyspnea. In total, 18 diagnostic and 22 treatment options were offered to the participants. The order of the appearance of the diagnostic and treatment options in the case report was chosen randomly in order to avoid influencing physicians’ answers.

The MDs were asked to rank their diagnosis plans by writing numbers next to the 18 options. If the participants did not consider one or more of the given diagnostic options at all, the field next to the option was left blank. The same procedure was performed with the treatment options. The default first-line investigations included reading the patient’s chart, clinical inspection, auscultation, and, optionally, performing a chest x-ray and measuring oxygen saturation. Before continuing with second- or third-line investigations, symptomatic treatment should be started. Assuming the participants would be familiar with the concept of first-line, second-line, and third-line investigations, as described in the literature, explanations on this issue were not given in the case report. There was no correct answer regarding diagnostic options recommended; hence, a couple of different answers were acceptable. Concerning the ranking, no order was considered as correct or incorrect, but the goal was to evaluate different responses of the participants. Therefore, descriptive methods to describe the attitudes of the participants, without scoring or judging them, were used.

Optimal pharmacologic treatment for this patient would include oral or parenteral opioids after first-line investigations to immediately alleviate the patient’s symptoms. In addition, non-pharmacological interventions, such as placing the patient in an upright position or opening a window, would ameliorate symptoms. The guidelines to give opioids for refractory dyspnea in cancer patients should be common medical knowledge. Hence, we included all treatment options described in the literature and evaluated which treatment options are known to medical doctors and whether they would be applied in a “real-life scenario.” It is assumed that young physicians in particular need to be regularly instructed in the management of refractory symptoms. Numerous guidelines do not find their way into clinical practice if “eminence-based” practice overrules evidence-based practice [27]. Therefore, guidelines that include opioids for refractory dyspnea in cancer patients should be regarded as common medical knowledge. Within this study, we listed all evidence-based diagnostic and treatment options described in the literature.

All data were collected at the University Hospital Krems. Part of the introductory phase for physicians in training in this tertiary hospital is a basic medical seminar. This compulsory seminar includes lectures about emergency medicine, ethics, pharmacology, law, the Critical Incident Reporting System (CIRS), and palliative care. Before the lecture on palliative care started, the physicians in training received the case report and were asked to read it and answer the questions. In addition, the questionnaire was distributed to senior physicians at the same medical institution.

For the present study, as no patient data were involved, assessment by and permission from an ethics committee were not required, as confirmed by the local ethics committee of Karl Landsteiner University of Health Sciences.

For the final analysis, we evaluated how often a diagnostic procedure or therapeutic option was chosen (frequency of chosen options). Additionally, we evaluated which diagnostic procedure or therapeutic option was ranked first, second, third, and so on (ranking of procedures or option).

### Explaining measurement of frequencies

If three MDs ranked auscultation first, while seven ranked it second, and eleven third, three fourth, and the rest of the MDs would not rank auscultation at all, the total number of frequencies for auscultation would be 24 MDs suggesting auscultation as an appropriate diagnostic option (*n* = 24).

### Explaining measurement of ranking

If 24 physicians would rank inspection first among the diagnostic procedures, 19 physicians would rank measuring oxygen saturation first among diagnostic procedures, nine physicians would rank reading the chart on rank 1, and the rest would not rank any diagnostic procedure, then inspection would be ranked as the most important diagnostic first-line procedure.

For the statistical analyses, absolute frequencies and percentages are reported as descriptive statistics. Not all respondents answered each question completely; therefore, the numbers that constituted the basis for the analysis are included in the reported response. To compare two different groups (i.e., senior physicians vs. physicians in training), Fisher’s exact test was used, and a Fisher-Halton-Freeman test was used to assess the comparison of three or more groups. These tests deliver reliable results, even with a few observations. Data analysis was performed using the statistical program Microsoft Office Excel (version 15.27) and IBM SPSS Statistics (version 27, Armonk, NY, USA). A *p* value ≤ 5% was considered statistically significant. To avoid an increasing error of the second type, no multiplicity corrections were made.

## Results

The questionnaire was distributed to 95 senior physicians and 30 physicians in training attending the basic medical seminar. The senior physicians included 38 internal medicine specialists, 12 pneumologists, 15 radiation oncologists, 10 general practitioners, and 20 anesthesiologists (*n* = 95). Eighty-two senior physicians ranked their diagnostic and therapeutic preferences for the case report (response rate 86%). In total, seven senior physicians had a diploma in palliative care. There was no significant difference in the answers of the senior physicians with a diploma in palliative care compared to those without. Of the 30 physicians in training, 24 (response rate 80%) ranked their diagnostic recommendations, while 23 (response rate 76%) indicated their therapeutic preferences concerning this case report.

### Diagnostic approaches

#### Ranking of diagnostic procedures

The senior physicians ranked inspection of the patient first among the diagnostic procedures, as it was chosen as the most important first-line investigation by 24 (29.27%) participants. Measuring oxygen saturation and reading the patient’s chart were both ranked first by 19 (23.17%) of the senior physicians, hence constituting the second most important diagnostic procedures (Supplementary Fig. 1). For the physicians in training, reading the chart constituted the most important first-line investigation, as it was ranked first by nine (37.5%) participants. Inspection and taking the patient’s history were both ranked first by five (20.83%) of the physicians in training, marking the second most important procedures for the physicians in training (Supplementary Fig. 2).

Investigation of electrolytes, performing an echocardiogram or a ventilation-perfusion scan, more blood work, a chest x-ray, or evaluation of D-dimer levels was never ranked first, either by the senior physicians or by the physicians in training. For further results, see Supplementary Figs. 1-2.

#### Frequency of diagnostic procedures

Significant differences between the senior physicians and the physicians in training were found for auscultation (trend toward significance), blood gas analysis, and measurement of oxygen saturation (Table 1). Auscultation was chosen by 95.1% of the senior physicians, whereas only 83.3% of physicians in training considered this option an important diagnostic tool in this situation (*p* = 0.076). Blood gas analysis was suggested by 61% of the senior physicians and 95.8% of the physicians in training (*p* = 0.001). Measuring oxygen saturation was chosen by 82.9% of the senior physicians and 62.5% of the physicians in training (*p* = 0.048).

**Table1:** Frequency of chosen diagnostic options—diagnostic option vs. level of training (senior physicians*physicians in training)

Diagnostic option	Senior physicians (*n* = 82) *n* (%)		Physicians in training (*n* = 24) *n* (%)		*p* value*
	No	Yes	No	Yes	
Auscultation	4 (4.9)	78 (95.1)	4 (16.7)	20 (83.3)	0.076
Bedside spirometry	82 (100)	0	24 (100)	0	n.a
Blood gas analysis	32 (39.0)	50 (61.0)	1 (4.2)	23 (95.8)	0.001
Blood pressure	69 (84.1)	13 (15.9)	23 (95.8)	1 (4.2)	0.183
Chest x-ray	51 (62.2)	31 (37.8)	10 (41.7)	14 (58.3)	0.1
Creatine kinase	61 (74.4)	21 (25.6)	20 (83.3)	4 (16.7)	0.426
CT scan	72 (87.8)	10 (12.2)	21 (87.5)	3 (12.5)	1.000
D-dimer	61 (74.4)	21 (25.6)	16 (66.7)	8 (33.3)	0.448
Dyspnea scale	69 (84.1)	13 (15.9)	23 (95.8)	1 (4.2)	0.183
ECG	35 (42.7)	47 (57.3)	9 (37.5)	15 (62.5)	0.814
Echocardiogram	75 (91.5)	7 (8.5)	24 (100)	0	0.346
Electrolytes	75 (91.5)	7 (8.5)	21 (87.5)	3 (12.5)	0.691
Inspection	22 (26.8)	60 (73.2)	8 (33.3)	16 (66.7)	0.608
More blood works	76 (92.7)	6 (7.3)	19 (79.2)	5 (20.8)	0.120
Oxygen saturation	14 (17.1)	68 (82.9)	9 (37.5)	15 (62.5)	0.048
Percussion	51 (62.2)	31 (37.8)	18 (75.0)	6 (25.0)	0.332
Read chart	23 (28.0)	59 (72.0)	4 (16.7)	20 (83.3)	0.301
Taking history	40 (48.8)	42 (51.2)	14 (58.3)	10 (41.7)	0.489
Ventilation-perfusion scan	79 (96.3)	3 (3.7)	23 (95.8)	1 (4.2)	1.000

*ECG* electrocardiogram, *n.a.* not applicable

^*^Fisher’s exact test was applied

Other options for diagnostic procedures did not show significant differences between the two groups. Concerning the senior physicians, 73.2% vs. 66.7% of the physicians in training acknowledged inspection as a diagnostic option (*p* = 0.608), while 37.8% of the senior physicians and 25% of the physicians in training would perform percussion during the physical examination (*p* = 0.332). Reading the patient’s chart was considered by 72% of the senior physicians and 83.3% of the physicians in training to be a useful diagnostic option for gaining additional information about the patient’s present condition (*p* = 0.301). For half of the physicians (51.2% of the senior physicians and 41.7% of the physicians in training), taking a brief history represented an appropriate option (*p* = 0.489), while 37.8% of the senior physicians and 58.3% of the physicians in training considered a chest x-ray a validated tool for diagnosis (*p* = 0.1). Measuring the level of the patient’s electrolytes was suggested by 8.5% of the senior physicians and 12.5% of the physicians in training (*p* = 0.691). Echocardiography was considered by 8.5% of the senior physicians and none of the physicians in training (*p* = 0.346). No participant chose bedside spirometry as a diagnostic option. The distribution of those few participants who considered a computed tomography (CT) scan as a diagnostic option was almost equal: 12.2% of the senior physicians and 12.5% of the physicians in training indicated this as a further expedient diagnostic tool (*p* = 1.000). Low proportions in both groups (3.7% of the senior physicians and 4.2% of the physicians in training) chose a ventilation-perfusion scan as an investigation (*p* = 1.000).

### Therapeutic approaches

#### Ranking of therapeutic options

Delivery of oxygen was chosen as the most important therapeutic approach by the senior physicians: 43 (52.44%) ranked this first as a first-line therapy. Improving the patient’s position was ranked first by 14 (17.07%) of the senior physicians, hence constituting the second important therapeutic procedure. The third important therapy ranked first by the senior physicians was providing psychological support, as indicated by 11 (13.41%) of the senior physicians (Supplementary Fig. 3). Among the physicians in training, 12 (50%) ranked the delivery of oxygen first, and four (16.67%) ranked the application of subcutaneous opioids first. Three (12.5%) of the physicians in training suggested providing psychological support first (Supplementary Fig. 4). For further results, see Supplementary Figs. 3-4.

#### Frequency of therapeutic options

##### Non-pharmacological treatment options

Providing psychological support to patients with acute dyspnea is one of the most relevant non-pharmacological treatment options, and 73.2% of the senior physicians and 73.9% of the physicians in training would choose this tool (*p* = 1). Improving the patient’s position was chosen by more than half of the senior physicians (65.9%) and only 30.4% of the physicians in training (*p* = 0.004). Using a fan to ameliorate the patient’s symptoms was chosen only by the senior physicians (17.1% vs. 0%, *p* = 0.036). Opening a window was selected by 29.3% of the senior physicians and 17.4% of the physicians in training (*p* = 0.299).

##### Pharmacological treatment options

Supplemental oxygen was the most important therapeutic approach chosen, with 92.7% of senior physicians and 95.7% of physicians in training ranking this as first-line therapy with no significant difference between the groups (*p* = 1). The second most frequent drug chosen was the application of a ß-2 agonist chosen by 28% of the senior physicians and 21.7% of the physicians in training (*p* = 0.606). Corticosteroids would be used by 25.6% of the senior physicians and by 43.5% of the physicians in training. (*p* = 0.122). Regarding opioids, intravenous application was chosen by 43.9% of the senior physicians and 21.7% of the physicians in training (*p* = 0.089), whereas subcutaneous application was chosen by 48.8% of the senior physicians and 78.3% of the physicians in training (*p* = 0.017). Oral application was an option for 9.8% of the senior physicians and 8.7% of the physicians in training (*p* = 1). Intravenous application of benzodiazepines was chosen by 24.4% of the senior physicians and 13% of the physicians in training (*p* = 0.392). Oral application of benzodiazepines was chosen by 7.3% of the senior physicians and 7.6% of the physicians in training (*p* = 1). The subcutaneous route was chosen by 2.4% of the senior physicians and none of the physicians in training (*p* = 1). Anticholinergic drugs were considered by 12.2% of the senior physicians and 4.3% of the physicians in training (*p* = 0.449). Blood transfusions, promethazine, nitroglycerine, heparin, diuretics, antibiotics, chlorpromazine, and methylxanthines were considered by far less than 10% of both groups (Table 2).

**Table2:** Frequency of chosen therapeutic options—therapeutic option vs. level of training (senior physicians*physicians in training)

Therapeutic option	Senior physicians (*n* = 82) *n* (%)		Physicians in training (*n* = 23) *n* (%)		*p* value*
	No	Yes	No	Yes	
Antibiotics	81 (98.8)	1 (1.2)	23 (100)	0	1
Anticholinergic drugs	72 (87.8)	10 (12.2)	22 (95.7)	1 (4.3)	0.449
Benzodiazepines i.v	62 (75.6)	20 (24.4)	20 (87.0)	3 (13.0)	0.392
Benzodiazepines p.o	76 (92.7)	6 (7.3)	21 (91.3)	2 (8.7)	1
Benzodiazepines s.c	80 (97.6)	2 (2.4)	23 (100)	0	*i*
Chlorpromazine	82 (100 =	0	23 (100)	0	n.a
Corticosteroids	61 (74.4)	21 (25.6)	13 (56.5)	10 (43.5)	0.122
Diuretics	78 (95.1)	4 (4.9)	22 (95.7)	1 (4.3)	1
Fan	68 (82.9)	14 (17.1)	23 (100)	0	0.036
Heparin	80 (97.6)	2 (2.4)	22 (95.7)	1 (4.3)	0.528
Improvement of position	28 (34.1)	54 (65.9)	16 (69.6)	7 (30.4)	0.004
Methylxanthines	81 (98.8)	1 (1.2)	22 (95.7)	1 (4.3)	0.392
Nitroglycerine	80 (97.6)	2 (2.4)	21 (91.3)	2 (8.7)	0.208
Open window	58 (70.7)	24 (29.3)	19 (82.6)	4 (17.4)	0.299
Opioids i.v	46 (56.1)	36 (43.9)	18 (78.3)	5 (21.7)	0.089
Opioids p.o	74 (90.2)	8 (9.8)	21 (91.3)	2 (8.7)	1
Opioids s.c	42 (51.2)	40 (48.8)	5 (21.7)	18 (78.3)	0.017
Oxygen	6 (7.3)	76 (92.7)	1 (4.3)	22 (95.7)	1
Promethazine	82 (100)	0	23 (100)	0	n.a
Psychological support	22 (26.8)	60 (73.2)	6 (26.1)	17 (73.9)	i
ß-2-agonists	59 (72.0)	23 (28.0)	18 (78.3)	5 (21.7)	0.606
Transfusions	82 (100)	0	22 (95.7)	1 (4.3)	0.219

*i.v.* intravenously, *n.a.* not applicable, *p.o.* orally, *s.c.* subcutaneously

^*^Fisher’s exact test was applied

## Discussion

The major goal of this study was to evaluate physicians’ attitudes toward diagnostic approaches and the treatment of severe acute dyspnea in a patient with advanced cancer. The main findings showed that evidence-based first-line therapy with opioids was not the first choice of experienced senior physicians or physicians in training. Both groups of MDs ranked oxygen therapy and betamimetics as first-line treatment options. Furthermore, comparing the choices of different diagnostic options between the groups revealed a significant difference for the use of blood gas analysis (*p* = 0.01), measuring oxygen saturation (*p* = 0.048), and a trend toward a difference for auscultation.

In severely ill patients, appropriate symptom alleviation is the cornerstone of good medical care, and diagnostic procedures should always be accompanied by a consideration of their clinical consequences. Auscultation was chosen by almost all the senior physicians, whereas only about 80% of the physicians in training considered this to be an important diagnostic tool (*p* = 0.076). Percussion was chosen rarely by both groups (37.8% of senior doctors vs. 25.0% of physicians in training, *p* = not significant). Other first-line investigations did not show any significant differences between the two groups. The measurement of oxygen saturation is often used in the assessment of dyspnea, but it is of limited value [5]. However, 82.9% of the senior physicians, but only 62.5% of the physicians in training, would choose this option (*p* = 0.01). Second-line and third-line investigations were only rarely chosen by both groups.

Our study also showed differences in the ranking and use of therapeutic options. Whereas the treatment of pain with opioids has become routine not only for doctors familiar with the concepts of palliative care, dyspnea in patients with advanced cancer or other palliative care situations remains difficult. The restrained application of opioids in patients with refractory dyspnea in a palliative setting is often based on physician-based concerns about respiratory depression [28]. The attitudes of applying opioids to patients at the end of life were surveyed by Borasio et al. in 411 medical directors of neurological departments in Germany. Their results revealed that 32% thought that it was illegal to administer analgesics in doses that risk respiratory depression, and 45% of the neurologists believed that treating terminal dyspnea with morphine was equivalent to euthanasia [29]. A French study asked 791 general practitioners and oncologists whether they would prescribe morphine as a first-line therapy to patients with terminal lung cancer suffering from dyspnea associated with cough and great anxiety. Only half of the oncologists and 40% of the general practitioners stated that they would prescribe morphine in this situation. The attitude of prescribing opioids correlated with the physician’s age, professional background, communication skills, and attitudes toward terminally ill patients [30]. In our evaluation, 9.5% of all the MDs would apply opioids orally, 55.2% subcutaneously, and 39% intravenously in the presented case of a patient with advanced lung cancer and refractory dyspnea.

Altogether, the management of dyspnea in terminally ill patients might often be inadequate [29, 30]. Even in opioid-naive patients, there is no higher risk of respiratory depression or increase of pCO_2_ [31, 32]. In addition to non-pharmacological therapies, the only validated treatment for alleviating patients’ dyspnea is opioids administered either orally or parenterally [7, 33]. Till date, no data support the assumption that the use of opioids for dyspnea management is associated with a reduction in the patient’s life expectancy. On the contrary, patients who receive appropriate symptom management may have prolonged survival due to a reduction in physical and psychological stress and exhaustion The adverse effects of opioids, such as sleepiness, hypercapnia, or nausea, are very infrequent in patients with advanced cancer, and the occurrence of transient sedation after application of opioids may also be related to sleep deprivation due to uncontrolled dyspnea [21]. Till date, there are no controlled trials to compare the efficacy of various agents, routes of administration, the starting dose, and the optimal dosage using opioids against dyspnea in cancer patients. A few controlled trials with low sample sizes studied the use of morphine in cancer patients, administered orally, subcutaneously, intravenously, intramuscularly, or nebulized [34–38]. Thus, it remains unclear which opioid is most effective and whether there are differences between the agents. Large randomized clinical trials are needed to evaluate the optimal starting dose and the best mode of application of opioids [5]. Interestingly, in our study, the physicians in training would apply opioids subcutaneously significantly more often than the senior physicians (*p* = 0.017).

Next to opioids, our study also explored the use of additional pharmacologic treatment options. Although no data support the use of bronchodilators (e.g., β-2 agonists) as a first-line treatment, 28 of the physicians in training chose this option, maybe by assuming a bronchospastic component as an explanation of the patient’s dyspnea. Another explanation could be that physicians in training are less reluctant to use a bronchodilator than an opioid. Regarding benzodiazepines, 32.5% of all the participating MDs would apply these drugs in the given scenario. Although recent research has concluded that midazolam as an upfront therapy might be beneficial for patients, there is no overall benefit of benzodiazepines in reducing dyspnea in this patient population [8, 39, 40]. However, a Cochrane review recommended the use of benzodiazepines only if first-line treatment has failed [39].

Oxygen as the initial therapeutic approach was ranked first in both groups and was among the treatment options chosen by 93.3% of all the MDs. Two randomized studies compared the effects of supplemental oxygen and ambient air on dyspnea in patients with advanced cancer. When compared to ambient air in hypoxemic cancer patients at rest, supplemental oxygen significantly increased oxygen saturation [21]. Another trial by Booth et al. reported that ambient air was just as effective as oxygen in relieving dyspnea [41]. Until now, there have been no consensus guidelines on the use of supplemental oxygen for dyspneic cancer patients, but it appears reasonable to apply this option in dyspneic cancer patients with hypoxemia.

Finally, a previous study evaluated the attitudes of fourth-year medical students toward diagnostic and therapeutic approaches in a similar situation. Among the 423 participants, 92% considered oxygen the most important treatment option. However, 32.6% would also suggest the use of opioids as an option, which is comparable to our study results [42].

## Conclusion

The use of opioids as a first-line pharmacological treatment for cancer patients with severe dyspnea is recommended in recent guidelines [8]. Our study revealed that opioids to alleviate dyspnea in this scenario were only an option for less than half of the participating physicians.

Most physicians know that opioids may depress respiration. A reduction in the sensitivity and responsiveness of the medullary respiratory centers to hypoxia and hypercapnia could be one of the mechanisms explaining the respiratory depressant effect [43]. Opioids are known to reduce minute ventilation and decrease the tidal volume [44]. However, not only physicians in training but also senior physicians should be taught that there is no evidence for respiratory depression when carefully using opioids to manage dyspnea-related symptoms. Chronic ventilatory failure is neither common nor clinically significant in advanced cancer patients [45].

When this study took place, the study center had no evidence-based policy about how to assess and treat dyspnea in patients with advanced lung cancer and dyspnea. Based on the results of this study, we became aware of how heterogeneous the approach to the management of dyspnea in advanced disease is. We therefore developed an evidence-based Standard Operating Procedure at our institution, which is regularly updated. We also started to offer regular training, especially for young colleagues.

Our study results confirm the need for proper education of physicians in the diagnosis and management of dyspnea in cancer patients. The management of dyspnea might sound complex, but implementing a comprehensive assessment, discussing goals of care, and applying appropriate pharmacologic and non-pharmacologic interventions may positively impact the quality of life for patients with dyspnea in a palliative care setting.

## Supplementary Information

Below is the link to the electronic supplementary material.

Supplementary file1 (DOCX 173 KB)

## Data Availability

The datasets used and/or analyzed during this study are available from the corresponding author on reasonable request.

## Abbreviations

CT: Computed tomography
CIRS: Critical Incident Reporting System
MDs: Medical doctors
UICC: Union for International Cancer Control

## Code availability

Data analysis was performed using the statistical program Microsoft Office Excel (version 15.27) and IBM SPSS Statistics (version 27, Armonk, NY, USA).
