# Supplementary material for: Evaluation of diagnostic and treatment approaches to acute dyspnea in a palliative care setting among medical doctors with different educational levels
Source: Support Care Cancer. 2022 Mar 25;30(7):5759–68. doi: 10.1007/s00520-022-06996-6 (PMC9135814; doi:10.1007/s00520-022-06996-6)
Supplement: Supplementary file 1 — Supplementary file1 (DOCX 173 KB) [file 520_2022_6996_MOESM1_ESM.docx]

**Appendix / Supplementary**

**Patient case report**

Dear Participant,

Thank you for your cooperation in filling out the patient case report. You will be asked to read the case report of the patient and then give your opinion about diagnostic procedures and treatment options by putting numbers in a ranked manner next to the square. If you feel that you would not perform one of the procedures or would not use one of the treatment options, please leave the place next to the square blank.

**Example of how to complete the patient cases:**

To bake a cake, you will use some of the listed ingredients. Please rank ingredients in order of usage by placing a number next to the square. If you do not use one of the ingredients, please do not write a number next to the square:

Eggs Milk Flour Butter Sugar

Mud Baking powder Poison

If you bake the cake in the following way – eggs, sugar, butter, flour, baking powder, and milk without mud and poison, you will place the numbers next to the squares in this manner:

**1**  Eggs **6**  Milk **4**  Flour **3**  Butter **2**  Sugar
 Mud **5**  Baking powder Poison

If you bake the cake in the following way – flour, baking powder, milk, mud, eggs, butter, and sugar without poison, you will place the numbers next to the square in this manner:

**5**  Eggs **3**  Milk **1**  Flour **6**  Butter **7**  Sugar **4**  Mud

**2**  Baking powder Poison

**Appendix cont.**

**Patient case - Acute Dyspnea-Solid Tumor**

Clinical example

A 62-year-old man was diagnosed with adenocarcinoma of the lung six months ago. He also has a history of COPD GOLD II and arterial hypertension. The patient had smoked 20 cigarettes every day for 48 years of smoking history. Staging revealed Stage IV disease; at time of diagnosis, hepatic and pulmonal metastases were present. The patient received four cycles of chemotherapy with cisplatin and vinca alkaloids. Partial remission could be observed. He was referred to the hospital because of worsening dyspnea. A CT acan revealed massive progression of the lung metastases. Current medications included omeprazole 40 mg p.o.d, aspirin 100 mg p.o.d, and lisinopril 2.5 mg p.o.d. At 3 a.m., you are paged by the nurses because the patient is suffering from acute worsening of dyspnea.

Your diagnosis plan includes: (please mark your choices in a ranked manner. If you do not choose one of the options, please do not write a number next to the square):

Diagnostic options

Oxygen saturation Read patient’s chart quickly Percussion

Electrolytes ECG Inspection

Creatine kinase Ventilation-perfusion scan Blood gas

Echocardiogram CT scan More blood work

Auscultation Chest x-ray Bedside spirometry

Taking patient’s history D-dimer Dyspnea scale

Flow volume loop

After examining the patient, your assumption is that the dyspnea is related to the progression of lung metastases.

Your treatment plan includes: (please mark your choices in a ranked manner. If you do not use one of the options, please do not write a number next to the square):

Therapeutic options

Psychological support Oxygen Fan Opioids i.v.

Open window Opioids p.o. Benzodiazepines p.o.

Opioids s.c. Improve position Diuretics Transfusions
 Chlorpromazine Corticosteroids Beta-2-agonists

Nitroglycerine Anticholinergic drugs Promethazine

Antibiotics Heparin Benzodiazepines i.v

Methylxanthines Benzodiazepines s.c.

s.c.: subcutaneously; p.o.: per os; i.v.: intravenously.

Supplementary Figure 1. Frequency of diagnostic approaches as suggested by the senior physicians


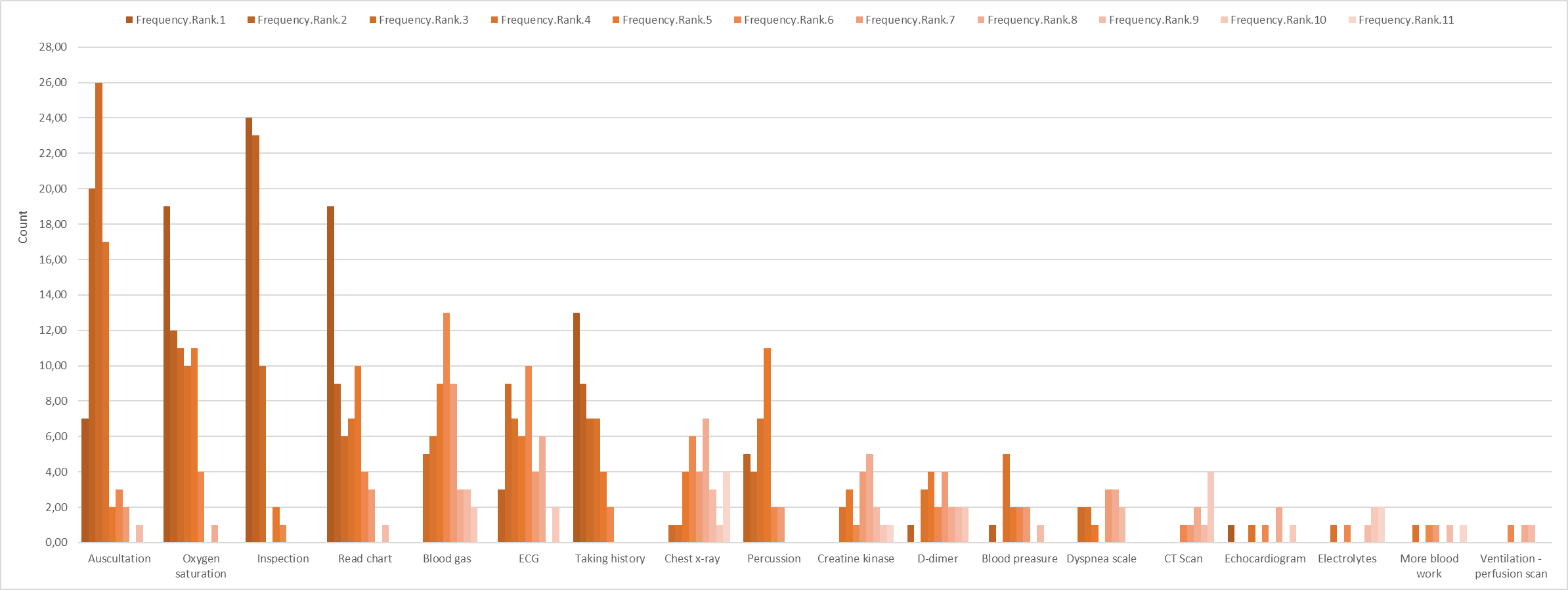


CT: computed tomography; ECG: electrocardiogram.

Supplementary Figure 2. Frequency of diagnostic approaches as suggested by the physicians in training


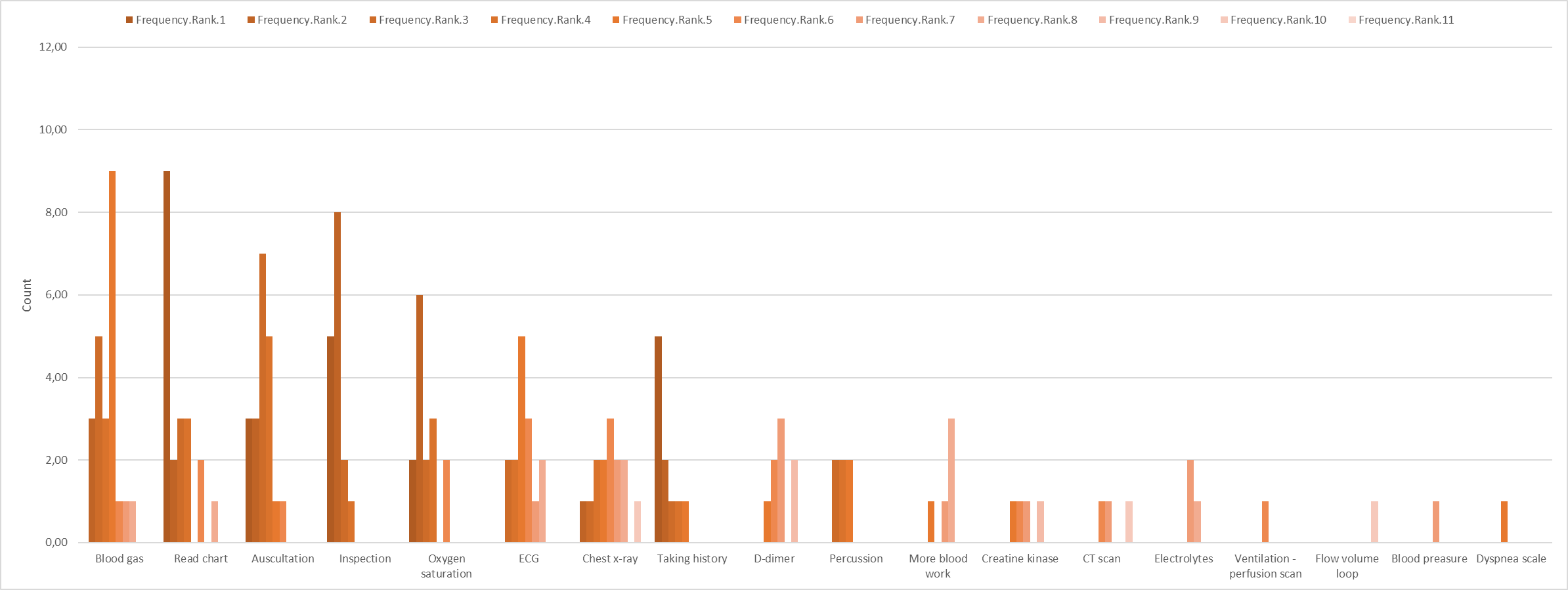


CT: computed tomography; ECG: electrocardiogram.

Supplementary Figure 3. Frequency of therapeutic approaches as suggested by the senior physicians


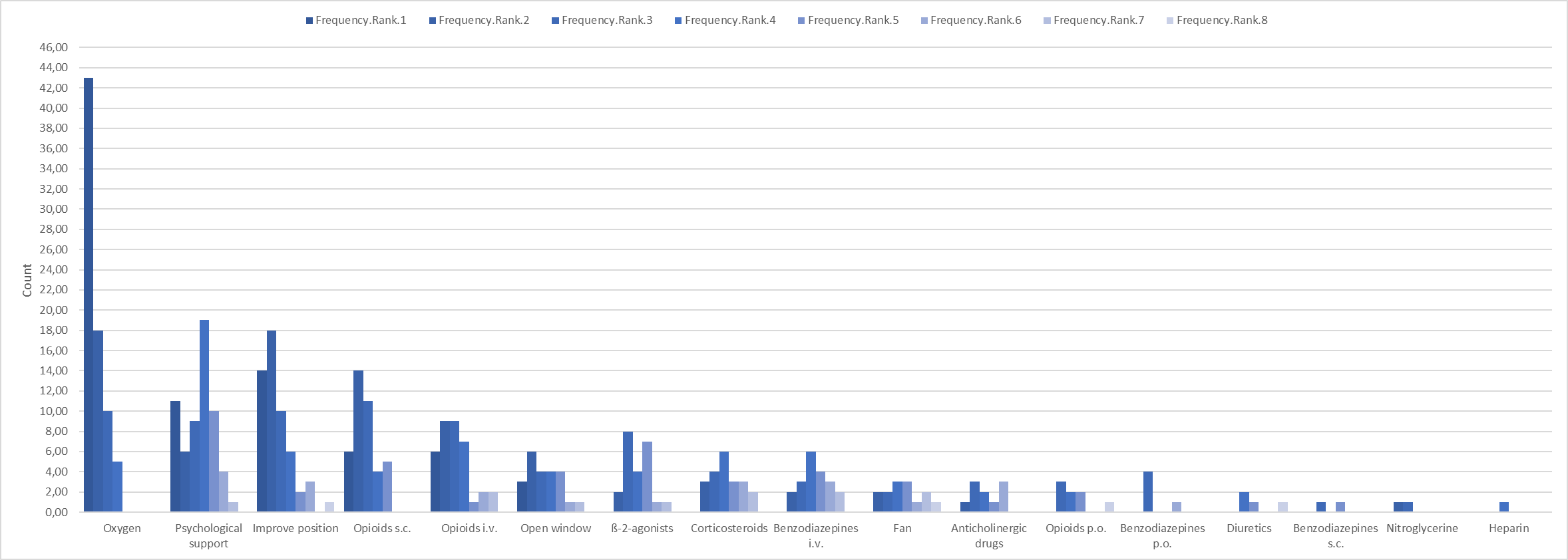


i.v.: intravenously; p.o.: orally; s.c.: subcutaneously.

Supplementary Figure 4. Frequency of therapeutic approaches as suggested by the physicians in training


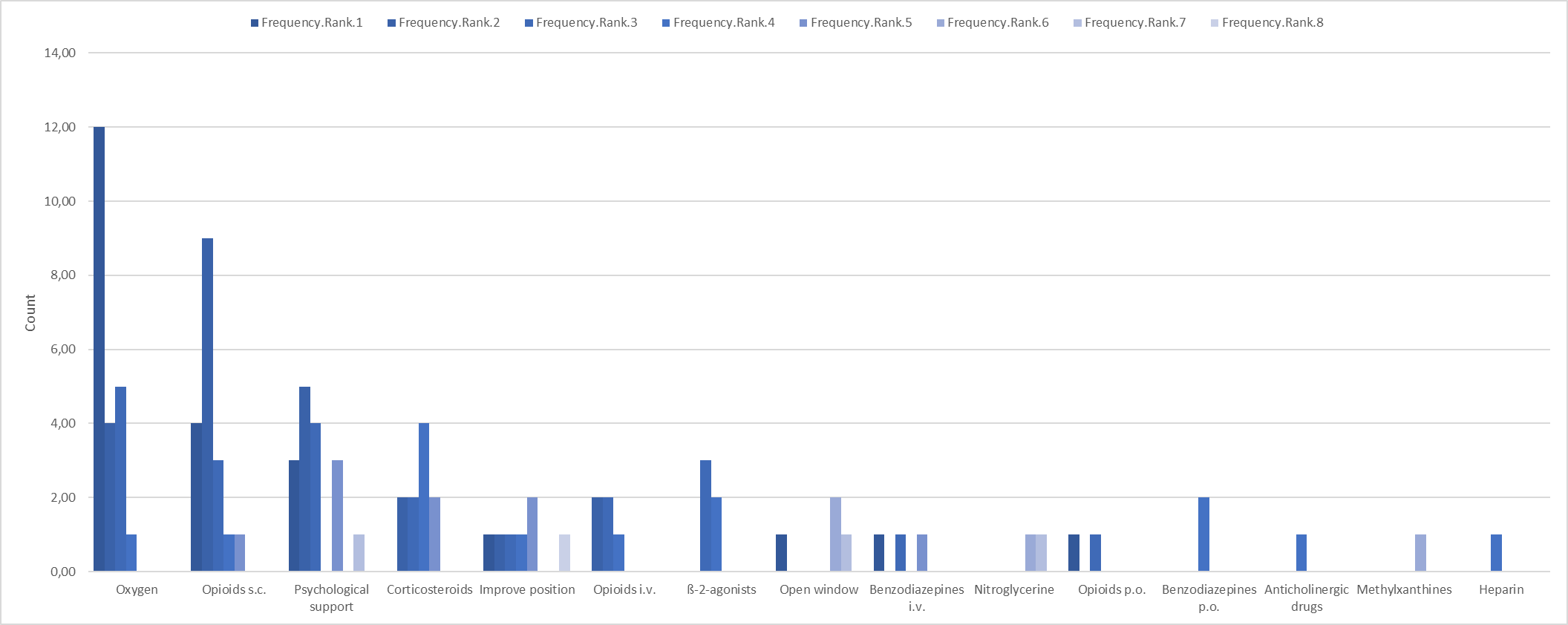


i.v.: intravenously; p.o.: orally; s.c.: subcutaneously.
